# Two-step pragmatic subgroup discovery for heterogeneous treatment effects analyses: perspectives toward enhanced interpretability

**DOI:** 10.1007/s10654-025-01215-y

**Published:** 2025-03-04

**Authors:** Toshiaki Komura, Falco J. Bargagli-Stoffi, Koichiro Shiba, Kosuke Inoue

**Affiliations:** 1 Department of Social and Behavioral Sciences, Harvard T. H. Chan School of Public Health, Boston, MA USA; 2 Department of Biostatistics, Fielding School of Public Health, University of California, Los Angeles, USA; 3 Department of Biostatistics, Harvard T. H. Chan School of Public Health, Boston, MA USA; 4 Department of Epidemiology, School of Public Health, Boston University, Boston, MA USA; 5 Department of Social Epidemiology, Graduate School of Medicine, Kyoto University, Floor 2, Science Frontier Laboratory, Yoshida-konoe-cho, Sakyo-ku, Kyoto, 604-8146 Japan; 6 Hakubi Center for Advanced Research, Kyoto University, Kyoto, Japan

**Keywords:** Heterogeneous treatment effect, Causal machine learning, Scientific communication, Diabetes, Cardiovascular events

## Abstract

Effect heterogeneity analyses using causal machine learning algorithms have gained popularity in recent years. However, the interpretation of estimated individualized effects requires caution because insights from these data-driven approaches might be misaligned with the contextual needs of a human audience. Thus, a *practical framework* that integrates advanced machine learning methods and decision-making remains critically needed to achieve effective implementation and scientific communication. We introduce a 2-step framework to identify characteristics associated with substantial effect heterogeneity in a practically relevant format. The proposed framework applies distinct sets of covariates for (i) estimation of individualized effects and (ii) subgroup discovery and shows the subgroups with heterogeneity based on highly interpretable if-then rules. By referring to existing metrics of interpretability, we describe how each step contributes to leveraging a theoretical advantage of machine learning models while creating an interpretable and practically relevant framework. We applied the pragmatic subgroup discovery framework for the Look AHEAD (Action for Health in Diabetes) trial to assess practically relevant and comprehensive insights into the effect heterogeneities of intense lifestyle intervention for individuals with diabetes on cardiovascular mortality. Our analysis identified (i) individuals with history of cardiovascular disease and myocardial infarction had the least benefit from the intervention, while (ii) individuals with no history of cardiovascular diseases and HbA1c < 7% received the highest benefit. In summary, our practical framework for heterogeneous effects discovery could be a generic strategy to ensure both effective implementation and scientific communication when applying machine learning algorithms in epidemiological research.

**Supplementary Information:**

The online version contains supplementary material available at 10.1007/s10654-025-01215-y.

## Introduction

There is a growing development and interest in estimating heterogeneous treatment effects (HTE) in epidemiologic research. While conventional subgroup analyses assess HTE based on one or two characteristics, modern non-parametric machine learning algorithms allow us to directly estimate treatment effects conditional on a multi-dimensional set of covariates [1]. Such estimates are known as conditional average treatment effects (CATEs) defined as $\:E\left[{{Y}_{i}}^{A=1}-{{Y}_{i}}^{A=0}|{X}_{i}={x}_{i}\right]$, where *i* is each individual, *Y* is the potential outcome, *A* is the binary treatment, and *x* is vector of covariates. By incorporating various interactions of treatment and covariates simultaneously in the model, these approaches (e.g., causal forest, Bayesian causal forest, other meta-learners) enable efficient discovery of complex and comprehensive insights into HTE assessment [2, 3]. In clinical research, HTE powered by machine learning is often described in the context of personalized treatment regimens, including effect score analysis and high-benefit approach [4–6]. In population health research, assessing HTE could also be important from the health equity perspective [7]. Thus, HTE analyses using machine learning play a critical role in advancing scientific knowledge and effective decision-making in public health practice.

At the same time, these data-driven approaches hold a fundamental dilemma; the flexible nature of non-parametric models that characterize their theoretical advantages might misalign them from practical decision-making. That is, non-parametric models are a powerful tool to disentangle the complexity of HTE through the estimation of CATEs (e.g., as a non-linear function of cardiovascular disease (CVD) risk factors including smoking, diabetes, and hypertension). However, statistical excellence does not always match with the decision-making process in practice for some reasons. First, the information constructing the underlying heterogeneity is not guaranteed to be also interpretable; for example, even if propensity score contributes to the substantial difference in CATE, decision-making directly based on propensity score is not practical. Furthermore, understandable or relevant characteristics are contextual to the audience and often informed by subject-matter knowledge [2]. Second, human audiences tend to seek a simpler explanation [8, 9]. In fact, decision-making in healthcare practice leverages existing thresholds constructed with simple rules (e.g., initiate high-intensity statin for individuals with 10-year CVD risk ≥ 20%) [10, 11]. Consequently, decision-making solely based on machine learning is not common in public health practice to date [12].

Although it is true that pure scientific inquiry in exploratory analysis could discover insights that might advance our knowledge, careful considerations about the pragmatic needs of the audience deserve more attention because the readers of clinical and epidemiologic journals are often healthcare professionals whose views might stem from existing practice. Conceptually, no superiority or inferiority should exist between these perspectives because it is a matter of the study’s objective and target audience, which is not solely based on a statistical standpoint. Their HTE insights could rather be complementary to each other. However, without harmonization with the context of its application, HTE analyses with machine learning might trigger confusion and even skepticism, which might hinder the constructive accumulation of scientific knowledge [10]. Thus, amid the development of statistical methods, the practical framework, with which applied researchers could embrace the contextual needs of the study objective, is critically needed. Such an implementation strategy with improved accountability will merit the credibility of applied analysis and enhance scientific communication [13].

The present study aims to propose a new framework of HTE analysis in epidemiologic research. We first introduce metrics to examine the interpretability of HTE analysis based on the predictive, descriptive, relevant (PDR) framework proposed by Murdoch et al. [14]. We then describe a 2-step approach to identify interpretable subgroups using decision trees in post-hoc [2, 15, 16]. The 2-step procedure enables researchers to (i) fully leverage input data without reducing or modifying information, (ii) discover and synthesize subgroups based on subject-matter knowledge, and (iii) critically examine potential gaps between data-driven insights and practical relevance.

## Methods

Table 1 summarizes the overview of each step in our framework—pragmatic subgroup discovery. We also demonstrate an applied example of pragmatic subgroup discovery using registered data from the Look AHEAD (Action for Health in Diabetes) trial [17].

**Table 1:** The overview of pragmatic subgroup discovery

	Step 1: CATE Estimation	Step 2: Subgroup Discovery
Purpose	- Estimate conditional average treatment effects (CATE) at any level of granularity.	- Identify subgroup with effect heterogeneity.
Model	- Causal forest, Bayesian causal forest, and other meta-learners.	- Classification and regression trees (CART) and other tree-based models.
Covariates	- Covariates can be used in *any format*. - Researchers might decide to perform feature engineering to enhance model fit if needed.	- Only select covariates useful for the decision-making related to the study objective. - Researchers might decide to convert continuous variables into categories, or even exclude from the covariate set.
Metrics of Interpretability	- Predictive accuracy^a^	- Descriptive accuracy^b^ - Relevancy^c^
Techniques	- Qini curve and coefficient, C-for-benefit, best predictor analysis, sample splitting, rank-consistency test, etc.	- Calibration test with regression models, sample splitting, etc.

Abbreviation: CATE, conditional average treatment effect. ^a^Predictive accuracy: the degree to which the statistical model accurately captures the relationships that researchers aim to understand (i.e., the estimation of heterogeneity in the treatment effect within the counterfactual framework). Such accuracy can be assessed by calibration tests, external data, or a cross-fitting approach. ^b^Descriptive accuracy: the extent to which the interpretation of the results accurately reflects the relationships computed from statistical models. ^c^Relevancy: the insights derived from statistical models are described in a manner that the human audience/decision-makers can reasonably understand

### PDR framework of interpretability

The PDR framework defines the interpretability of machine learning models by 3 metrics: (i) predictive accuracy, (ii) descriptive accuracy, and (iii) relevancy [14]. First, *predictive accuracy* refers to the degree to which the statistical model captures the relationships that researchers aim to understand [14]. Second, *descriptive accuracy* is defined as the extent to which the interpretation of analysis results objectively reflects the relationships computed from statistical models [14]. Third, *relevancy* indicates that the insights derived from statistical models are described in a manner that the human audience/decision-makers can reasonably understand [14]. Thus, in the context of HTE analysis with enhanced interpretability, researchers anticipate (i) a statistical model to capture the underlying heterogeneities in the counterfactual framework (predictive accuracy), (ii) the interpretation method to unfailingly summarize the trends of heterogeneity derived from the model (descriptive accuracy), and (iii) the discovered heterogeneities to be presented in a practically relevant format (relevancy).

### Step1: CATE estimation

The first step is the estimation of CATEs. The aim of this step is to let the chosen statistical model explore the complexity of the input data through the estimation of CATEs. Any modeling approac could be applied in this stage; for example, meta-learners, including causal forest and Bayesian causal forest, are popular methods in CATE estimation [2, 3]. As each modeling has its own strengths and weaknesses, researchers might decide which models could be most suitable in the given dataset and domain knowledge. Details and comparisons of meta-learners can be found elsewhere [18–20]. Most importantly, this step of CATE estimation can be done using covariates in any format. For example, researchers can retain a continuous covariate in the model as is rather than categorizing it to model a non-linear relationship. In these cases, the modified covariates might not be in a directly interpretable form. The key idea is to guide the chosen model to capture the underlying heterogeneity through a reasonable model fit to achieve the first metric of interpretability—predictive accuracy. One could describe this model fitting process as a “model-driven” estimation of CATEs.

To assess potential overfitting and the failure of convergence in the statistical model, it is crucial to examine whether the model fits the input data successfully, although some algorithms inherently have features to minimize overfitting, such as splitting samples into training and test data (i.e., honesty), cross-fitting, and Bayesian regularization [2, 21, 22]. Here we introduce some techniques to assess the calibration performance. First, the Qini curve exhibits the frequency of outcome in the treatment arm compared with the control arm which was estimated to have similar levels of CATEs [23]. The x-axis sorts all samples from lowest to highest estimated CATEs and shows the proportion of the samples (from 0 to 100%). The y-axis represents the cumulative difference in the outcome occurrence between treatment and control arms. With a reasonable calibration performance of CATE, we anticipate that the cumulative difference to be higher than in a scenario where samples are sorted at random. The Qini coefficient quantifies this difference between the Qini curve and the random ordering. Additionally, C-for-benefit is the statistic that represents the probability of concordance between the predicted and observed benefit in samples [24]. The Qini coefficient > 0 and C-for-benefit statistic > 0.5 provide evidence of calibration that is better than by chance, respectively [25]. Alternative approaches for the calibration test include best linear predictor analysis and non-parametric rank-consistency test [6, 21, 22, 26].

### Step2: subgroup discovery

The second step aims to identify subgroups characterized by a set of interpretable variables. In this paper, we refer to these covariates as ***interpretable covariates***. While the first step primarily focuses on fitting a statistical model to the input data to estimate the CATE, in this stage, researchers synthesize interpretable covariates by selecting and defining covariates in a way where the identified subgroups are reasonably related to practical decision-making. For example, 3 age subgroups (e.g., < 40, 40–59, and ≥ 60) could be more helpful for clinicians to consider certain treatment options for patients with diabetes, compared to proposing somewhat arbitrary age thresholds determined based on a purely data-driven analysis of a continuous age. In such a case, researchers might choose to include pre-specified age subgroups in interpretable covariates rather than using age on a continuous scale. The selection and modification of covariates in this step are important because if researchers choose to remove or reduce the information in CATE estimation, statistical models might fail to incorporate information that was contributing to the underlying heterogeneity, limiting the predictive accuracy. Of note, researchers might apply distinct classifications of variables for different study objectives even if they use an identical dataset. Moreover, when some covariates such as propensity score for the treatment could be informative but challenging to connect to a practical sense, one might exclude them from a list of interpretable covariates. The selection of interpretable covariates could be considered as a reduction of data complexity driven by principled and subject-matter knowledge to ensure the last element of interpretability—relevancy.

One example of simple approaches to identifying subgroups using interpretable covariates is classification and regression trees (CART) using the estimated CATEs [2, 27]. CART is an algorithm that divides data into subgroups by creating decision trees. In each split of input data, CART recursively synthesizes criteria that maximize the homogeneity of a targeted value [27]. In our discovery framework, CART divides samples into subgroups based on if-then rules, which resemble human decision-making processes, using the estimated CATE and chosen set of covariates [15]. The identified subgroups are defined with mutually exclusive sets of characteristics; for example, if gender and age were applied, CART might divide samples into (i) male & age < 40, (ii) male & ≥40, (iii) female & age < 40, and (iv) female & age ≥ 40) [28]. Researchers can control the number of criteria used to characterize subgroups by setting the depth of the splitting in CART; the higher depth of data splitting results in subgroups defined with more covariates. The direct use of CATE estimates from the chosen statistical models in CART helps to enhance the second metric of interpretability—descriptive accuracy. By sorting CATE with interpretable covariates, this step integrates the estimates derived from statistical modeling with practically meaningful classifications without limiting the roles of each. Alternatively, one can apply other tree models to synthesize subgroups [15, 16, 29–32].

After subgroup discovery using CART, one can assess whether the computed decision rules reasonably reflect the effect heterogeneity by estimating the ATE of each subgroup by separate regression models. Code examples are provided on GitHub (https://github.com/Toshi934/Interpretability/blob/main/Simulation.R).

### Example: pragmatic subgroup discovery in the Look AHEAD trial

In this section, we demonstrate the practical utility of the proposed two-step framework for pragmatic subgroup discovery using registered data of the Look AHEAD trial [17]. In brief, Look AHEAD is a randomized trial of individuals with diabetes to compare intensive lifestyle intervention and diabetes support and education [17]. After treatment assignment, participants were followed up for 13.5 years (median follow-up: 9.6 years) [17]. We obtained 4,901 individuals from the Look AHEAD trial from the National Institute of Diabetes and Digestive and Kidney Diseases Repository. From 4,901 individuals, we excluded 304 individuals (6.2%) who were lost to follow-up before developing the outcome within 7 years, resulting in 4,597 analytic samples. Supplementary Fig. 1 illustrates the sample selection flow.

### Intervention

Individuals with type 2 diabetes were randomly assigned to either intensive lifestyle intervention (treatment) or diabetes support and education (control). The treatment group received an intervention focused on weight loss through dietary management and physical activity.

### Outcome

The outcome of the present study was the trial’s primary outcome, including the first occurrence of death from cardiovascular causes, non-fatal myocardial infarction, non-fatal stroke, or hospitalization for angina. The outcome event within 7 years after the treatment assignment was assessed. In analysis, the outcome event was reverse coded so that a higher CATE indicates the higher benefit of treatment.

### Covariates

We used 47 covariates from the registered dataset. All covariates were used in CATE estimation. For subgroup discovery, we selected 42 interpretable covariates after categorizing and removing variables used in CATE estimation [33–41]. Table 2 summarizes covariates that were modified or removed in subgroup discovery. Of note, the manner of modification and removal might differ based on the purpose and audience of the analysis. See Appendix 1 for rationates of each definition. Missing data was imputed using a random forest via the package *missRanger* [42].

**Table 2:** Coding of interpretable covariates

Variable	Format Used in CATE Estimation	Format Re-coded for Subgroup Discovery
Age	Continuous	2 groups: < 60 or ≥ 60
BMI (kg/m²)	Continuous	6 groups: < 18.5, 18.5–24.9, 25-29.9, 30-34.9, 35-39.9, or ≥ 40
Alcohol Consumption (oz/week)	Continuous (oz/week)	2 groups: Drink Alcohol or Do Not Drink Alcohol
Fasting Glucose (mg/dl)	Continuous	3 groups: < 54, 54-69.9, or ≥ 70
Hemoglobin A1c (%)	Continuous	7 groups: < 5.7, 5.7–6.3, 6.4–6.9, 7.0-7.9, 8.0-8.9, 9.0-9.9, or ≥ 10
HDL Cholesterol (mg/dl)	Continuous	3 groups: < 40, 40-59.9, or ≥ 60
LDL Cholesterol (mg/dl)	Continuous	5 groups: < 100, 100-129.9, 130-159.9, 160-189.9, or ≥ 190
Triglycerides (mg/dl)	Continuous	4 groups: < 150, 150-199.9, 200-499.9, or ≥ 500
Urine Albumin (mg/dl)	Continuous	Removed
Urine Creatinine (mg/dl)	Continuous	Removed
Urine Albumin Creatinine Ratio	Continuous	3 groups: < 30, 30–299, or ≥ 300
Systolic Blood Pressure	Continuous	6 groups: < 120, 120-129.9, 130-139.9, 140-159.9, 160-179.9, or ≥ 180
SF-36 General Health	Continuous	2 groups: < 50 or ≥ 50
SF-36 Mental Health	Continuous	2 groups: < 50 or ≥ 50
SF-36 Bodily Pain	Continuous	2 groups: < 50 or ≥ 50
SF-36 Physical Functioning	Continuous	2 groups: < 50 or ≥ 50
SF-36 Role-Emotional	Continuous	2 groups: < 50 or ≥ 50
SF-36 Role-Physical	Continuous	2 groups: < 50 or ≥ 50
SF-36 Social Functioning	Continuous	2 groups: < 50 or ≥ 50
SF-36 Vitality	Continuous	2 groups: < 50 or ≥ 50
SF-36 Transition	Continuous	Removed
SF-36 Mental Component Summary	Continuous	Removed
SF-36 Physical Component Summary	Continuous	Removed
Beck Score of Depression	Continuous	4 groups: ≤ 10, 10–19, 20–29, or ≥ 30

Abbreviations: CATE, conditional average treatment effect; BMI, body mass index (kg/m²); HDL, high density lipoprotein (mg/dL), LDL, low density lipoprotein (mg/dL); and SF-36, 36 Item Short Form

### Statistical analysis

First, we applied a machine learning approach called Bayesian causal forest (BCF) to estimate CATEs of intensive lifestyle intervention on the reduction of primary outcomes on a scale of risk difference [RD] [2]. Details of BCF can be found in Appendix 2. Using all 47 covariates, we grew 300 regression trees for the 2 BART functions to construct a BCF model. We applied all samples to build the model to avoid potential variability in the results [43]. The model was trained through 300 iterations and 300 burn-in. To account for the potential selection bias due to the attrition within 7 years of follow-up, BCF was adjusted with inverse probability of censoring weighting (IPCW) conditional on the treatment and the 47 covariates [46]. To assess the calibration performance of the trained BCF model, we plotted the Qini curve and estimated the Qini coefficient and C-for-benefit.

Second, to identify subgroups associated with HTE, we built a CART model for predicting the estimated CATEs using the 42 interpretable covariates [27]. We performed 2 node splitting to characterize interpretable subgroups with 2 if-then rules based on their CATEs. To compare the result using interpretable covariates, we additionally performed the subgroup discovery using all 47 covariates without defining interpretable covariates (i.e., categorization). After subgroup discovery, we evaluated whether each interpretable subgroup reflects the effect heterogeneity identified in the first step. For simplicity, we computed group-specific ATE of the intervention via augmented inverse probability weighting (AIPW) using the estimated CATE in each interpretable subgroup.

## Results

Among 4,597 analytic samples, 2,309 individuals received the intervention and 2,288 were in the control group. The distribution of characteristics was well-balanced between the intervention group and the control group (Supplementary Table 1). Within the follow-up period of 7 years, 606 (13.2%) outcome events were observed. After training the BCF model, the Qini curve indicated a reasonable calibration performance (Supplementary Fig. 2). The Qini coefficient and C-for-benefit were 1.77 and 0.57 (95% CI = 0.54, 0.60), respectively. Supplementary Fig. 3 shows the distribution of the estimated CATE.

**Fig. 1:**
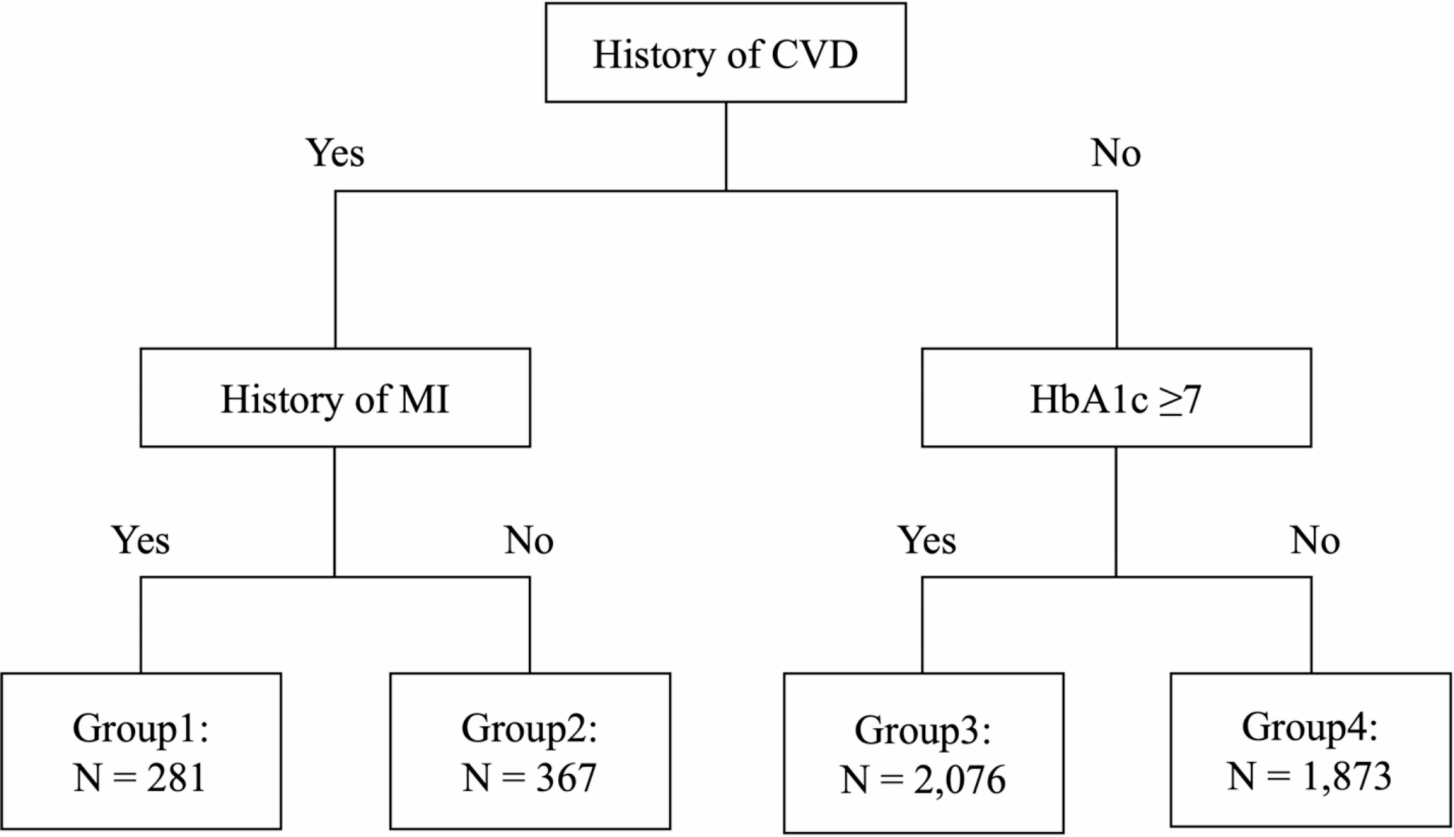
Subgroup discovery using conditional average treatment effects of intensive lifestyle intervention on cardiovascular outcomes by classification and regression tree. ^a^, Abbreviations: CVD, cardiovascular disease; MI, myocardial infarction; and HbA1c, hemoglobin A1c (%). ^a^ Classification and regression tree was created to predict the conditional average treatment effects using interpretable covariates. Conditional average treatment effects were estimated by the Bayesian causal forest algorithm

The CART model also indicated 4 subgroups characterized by 2 if-then rules based on interpretable covariates (Fig. 1). The CART indicated the subgroups had the lowest CATEs (i.e., the lowest benefit) in the following order: (i) individuals with history of CVD and myocardial infarction (MI) (group 1), (ii) those with history of CVD but MI (group 2), (iii) individuals with no history of CVD and HbA1c ≥ 7% (group 3), and (iv) individuals with no history of CVD and HbA1c < 7% (group 4). Results of AIPW analysis showed that subgroups with higher CATEs also had higher RDs (group 1: RD [95% CI] = -9.75pp [-20.98, 1.48]; group 2: RD [95% CI] = -0.57pp [-9.84, 8.70]; group 3: RD [95% CI] = -1.51pp [-3.99, 0.98]; and group 4: RD [95% CI] = + 3.24pp [0.81, 5.67]; Table 3). Supplementary Fig. 4 shows the results of subgroup discovery when all 47 covariates are used without selection and modification of covariates in step 2: although individuals with the lowest CATEs remained to be characterized with history of CVD and MI, the threshold of HbA1c (6.85%) indicated lower relevancy than that obtained from our framework.

**Table 3:** Absolute risk for cardiovascular outcomes after intensive lifestyle intervention among subgroups suggested by classification and regression tree^a, b^

Subgroup Suggested by CART	*N*	RD [95% CI]
All Sample	4,597	-0.64pp [-1.25, 2.53]
**Subgroup analysis**		
Group1 (History of CVD & History of MI)	281	-9.75pp [-20.98, 1.48]
Group2 (History of CVD & No History of MI)	367	-0.57pp [-9.84, 8.70]
Group3 (No History of CVD & HbA1c ≥ 7)	2,067	-1.51pp [-3.99, 0.98]
Group4 (No History of CVD & HbA1c < 7)	1,873	+ 3.24pp [0.81, 5.67]

Abbreviations: CART, classification and regression tree; RD, risk difference; CI, confidence interval; PP, percentage points; CVD, cardiovascular disease; MI, myocardial infarction; and HbA1c, hemoglobin A1c (%). ^a^Subgroups were determined by a single classification and regression tree. The classification and regression tree was created to predict the conditional average treatment effects estimated by the Bayesian causal forest algorithm. The greater conditional average treatment indicates the higher benefit from the intervention. ^b^ Risk differences were estimated via augmented inverse probability weighting using the conditional average treatment effects estimated by the Bayesian causal forest algorithm

## Discussion

The present paper described a 2-step framework in HTE analysis that discovered interpretable subgroups. In the applied example using the Look AHEAD trial, we first constructed a BCF model to estimate CATEs of the intervention on cardiovascular outcomes using all 47 covariates. In the second step, we identified subgroups with considerable effect heterogeneity via CART and 42 interpretable covariates. The results indicated individuals with a history of CVD and MI had the lowest benefit from the intervention compared to the control, and those with no history of CVD and HbA1c < 7% received the higher benefits. While machine learning algorithms have theoretical advantages in revealing intertwined effect modification and interactions between variables, effective scientific communications to the decision-making in a human audience often require a pragmatic reduction of data complexity. In this regard, our subgroup discovery framework in HTE analysis suggests the explicit distinction between the exploratory phase and the presentation of the results based on subject-matter knowledge. This could be a generic strategy in applied studies using machine learning algorithms to ensure the relevancy of the HTE insights while exploring the complex data structure.

Remarkably, although the original Look AHEAD study also highlighted history of CVD could be an important source of heterogeneity, their regression approach only allowed them to assess a single interaction between intervention and history of CVD [17]. In contrast, our 2-step framework uncovered further insights into the HTE of intensive lifestyle intervention compared to diabetes support and education by incorporating additional characteristics. The results indicate individuals with a history of CVD and MI received substantially lower benefits from the lifestyle intervention compared to other strata, providing more detailed and comprehensive evidence of heterogeneity.

Our subgroup discovery using all 47 covariates identified individuals with HbA1c < 6.85% have the highest level of benefit. Because this cut-off is more ambiguous than the threshold of HbA1c < 7% in our interpretable covariate, we believe the use of interpretable covariates contributed to the interpretation with improved relevancy. The discrepancies between these results indicate the level of descriptive accuracy in subgroup discovery; a more significant difference might indicate lowered descriptive accuracy in exchange for higher relevancy [14]. Such potential trade-off must be scrutinized based on the study objective. While using a selected subset of interpretable covariates could enable more effective scientific communications to health professionals, using all 47 covariates without categorizing continuous variables might be justified when the motivation is purely scientific discovery of HTE insights. In this context, our 2-step subgroup discovery framework could be useful to critically examine the connection between data-driven insights and communication toward the audience who could merit from the exploratory analysis.

Several important notes on the proposed framework should be acknowledged. First, researchers can create interpretable covariates in any format as long as they are relevant to the study objective. Even with the same dataset, researchers might decide to apply distinct variable selection and classifications for different study aims, such as clinical guidelines and policy decision-making. As such, researchers need to be critical about scientific communication; interpretable covariates should be coded in suitable formats for the audience who could benefit from the presented results. Second, while we employed CART for subgroup discovery to describe the framework with simplicity, other tree models, including optimal decision trees, can be applied [31, 32]. In fact, CART has several limitations. For example, the identified subgroups might not be globally optimal; characteristics detected by a single tree could be interpreted as jointly creating HTE but not as separate drivers [15, 31]. Moreover, the use of a single tree depending on the point estimate of CATE could lack stability. Causal rule ensemble could be an alternative subgroup discovery strategy to mitigate the concern associated with the use of a point estimate of CATE for each sample [15]. This algorithm creates many decision trees via bootstrap sampling and quantifies the uncertainty of synthesized subgroups. Other statistical considerations, including the sensitivity and loss to follow-up of samples, could be addressed through other methods [44–47]. Third, although we found evidence of heterogeneity in the Look AHEAD trial by comparing CATE subgroups, it is important to note that heterogeneity is inherently a relative concept. That is, to identify HTE in a given subgroup, one must establish a reference point for comparison [5, 15, 48], and clearly define how to assess HTE based on research questions and data structure. Fourth, existing literature might describe CATE in a more detailed manner. For example, the most granular level of CATE, characterized by various covariates, could be referred to as the individual (average) treatment effect, while noting an aggregate of CATEs by subgroups as the group average treatment effect [15, 22]. Researchers might choose to use these terminologies interchangeably, but clarity on the causal estimand of interest is critical. Lastly, we kept our applied example simple to prioritize the framework with clarity. However, future studies are needed to explore whether and to what extent the statistical details affect the result of the proposed framework, including the application of models other than BCF (e.g., causal forest, other meta-learners) and validation using external data.

Various machine learning algorithms have been proposed for HTE analysis in the past decades [18–20]. The semi- and non-parametric nature of these statistical modeling allows researchers to consider complex interactions between treatment and covariates without pre-specification. However, the direct interpretation of CATE, the estimand of these approaches characterized with various covariates, requires strong assumptions, and, therefore, imposes a challenge in practical use [49, 50]. As such, researchers attempted to extract characteristics significantly contributing to HTE [2, 15, 16, 51–57]. Subgroup discovery with decision trees showed promising interpretability by characterizing heterogeneity based on simple if-then rules, albeit these strategies might still derive arbitrary thresholds with less relevancy [2, 15, 28, 32]. However, this problem can be mitigated by creating dummy or categorical variables based on the threshold of interest, and using them as a candidate for the tree model. Our framework, which explicitly divides HTE analysis into data-driven and post-hoc interpretable steps with selected covariates, enhances the application of existing non-parametric modeling and achieves improved scientific communication with a human audience.

## Conclusions

We described a 2-step framework to integrate the advantages of data-driven methods with the contextual relevance of a human audience in HTE analysis. Our applied example using the Look AHEAD trial showed that individuals with no history of CVD and HbA1c < 7% benefited from intensive lifestyle intervention, while those with a history of CVD and MI received detrimental effects. Our discovery framework that explicitly distinguishes exploratory analysis and the presentation of its insights based on subject-matter knowledge could be a generic strategy to harness emerging advanced methodologies to effective scientific communication.

## Electronic supplementary material

Below is the link to the electronic supplementary material.

Supplementary Material 1

## Data Availability

The data that support the findings of this study are available from the National Institute of Diabetes and Digestive and Kidney Diseases Repository upon request.
