# Supplementary material for: Two-step pragmatic subgroup discovery for heterogeneous treatment effects analyses: perspectives toward enhanced interpretability
Source: Eur J Epidemiol. 2025 Mar 4;40(2):141–50. doi: 10.1007/s10654-025-01215-y (PMC12018488; doi:10.1007/s10654-025-01215-y)
Supplement: Supplementary file 1 — Supplementary Material 1 [file 10654_2025_1215_MOESM1_ESM.pdf]

Supplemental Appendix

Two-Step Pragmatic Subgroup Discovery for Heterogeneous Treatment Effects Analyses: Perspectives Toward Enhanced Interpretability

Appendix

1. Defining Interpretable Covariates
2. Bayesian Causal Forest

Supplementary Tables

1. Baseline Characteristics of Analytic Samples

Supplementary Figures

1. Sample Flow Chart
2. The Qini curve
3. Distribution of Conditional Average Treatment Effect of Intensive Lifestyle Intervention on Cardiovascular Events
4. Subgroup Discovery without Interpretable Covariates (All 47 Covariates)

Appendix 1. Defining Interpretable Covariates.

Age: Because the Look AHEAD trial had a specific eligibility criterion of age between 45 to 75, we categorized the age into middle age (<60) or older (≥60) [1].

BMI (kg/m^2): We applied the following six classifications of BMI among participants with diabetes (<18.5, 18.5-24.9, 25-29.9, 30-34.9, 35-39.9, and ≥40) [2].

Alcohol Consumption (oz/week): We converted the continuous value of alcohol consumption into a dichotomous indicator.

Fasting Glucose (mg/dl): We classified the level of fasting glucose following an existing standard of hypoglycemia diagnosis [3].

Hemoglobin A1c (%): The classification was determined by a combination of general criteria of diabetes diagnosis and an indicator for glycemic control [4,5].

HDL Cholesterol (mg/dl): An existing threshold of HDL levels was applied [6].

LDL Cholesterol (mg/dl): An existing threshold of LDL levels was applied [6].

Triglycerides (mg/dl): An existing threshold of triglyceride levels was applied [6].

Urine Albumin and Urine Creatinine (mg/dl): These values were removed from subgroup discovery because clinical assessment conventionally leverages their ratio rather than individual values.

Urine Albumin Creatinine Ratio: An existing threshold of albuminuria diagnosis was applied [7].

Systolic Blood Pressure: An existing classification of hypertension with levels of severity was applied [8].

SF-36 General Health: We converted the score into a dichotomous indicator of poor (<50) or good (≥50) self-reported conditions as a score of 50 indicates the normative value for all scales [9].

SF-36 Mental Health: We converted the score into a dichotomous indicator of poor (<50) or good (≥50) self-reported conditions as a score of 50 indicates the normative value for all scales [9].

SF-36 Bodily Pain: We converted the score into a dichotomous indicator of poor (<50) or good (≥50) self-reported conditions as a score of 50 indicates the normative value for all scales [9].

SF-36 Physical Functioning: We converted the score into a dichotomous indicator of poor (<50) or good (≥50) self-reported conditions as a score of 50 indicates the normative value for all scales [9].

SF-36 Role-Emotional: We converted the score into a dichotomous indicator of poor (<50) or good (≥50) self-reported conditions as a score of 50 indicates the normative value for all scales [9].

SF-36 Role-Physical: We converted the score into a dichotomous indicator of poor (<50) or good (≥50) self-reported conditions as a score of 50 indicates the normative value for all scales [9].

SF-36 Social Functioning: We converted the score into a dichotomous indicator of poor (<50) or good (≥50) self-reported conditions as a score of 50 indicates the normative value for all scales [9].

SF-36 Vitality: We converted the score into a dichotomous indicator of poor (<50) or good (≥50) self-reported conditions as a score of 50 indicates the normative value for all scales [9].

SF-36 Transition, SF-36 Mental Component Summary, and SF-36 Physical Component Summary: We removed these items because the above 8 scales represent the summary of self-reported health conditions [10–12].

Beck Score of Depression: We classified the level of depression following an existing threshold [13].

Abbreviations: BMI, body mass index (kg/m^2); HDL, high density lipoprotein (mg/dL), LDL, low density lipoprotein (mg/dL); and SF-36, 36 Item Short Form.

Appendix 2. Bayesian Causal Forest.

Bayesian causal forest (BCF) employs the framework defined as $Y_i = \mu(X_i) + \tau(X_i)A_i + \varepsilon_i$, where $\mu(X_i)$ denotes the mean untreated outcome (the prognosis) as a function of covariates, $E[Y_i|A_i = 0, X_i = x_i]$, $\tau(X_i)$ is the CATE as a function of covariates, and ε_i is an individual error term [14]. Based on the assumptions of conditional exchangeability given the set of covariates, consistency, and positivity, BCF estimates CATEs by finding nonparametric functions $\mu(X_i)$ and $\tau(X_i)$ which fit the training data well. More specifically, $\mu(X_i)$ and $\tau(X_i)$ are represented as sums of regressions trees defined as $f(x) = \sum_{k=1}^{K} f_k(x)$, where $f(x)$ is a regression tree function, and k is the number of trees [15]. BCF uses a Bayesian regularization approach that imposes a preference for simple decision rules, making it an extension of the well-known Bayesian additive regression trees (BART) model [15]. BART often shows high predictive abilities for out-of-sample prediction compared to other regression tree-based methods such as gradient boosting or random forests [16–18]. BART inference is conducted through Markov chain Monte Carlo (MCMC) [15]. BCF was implemented using the R package *bcf* [19].

Reference

1. Look AHEAD Research Group, Wing RR, Bolin P, Brancati FL, Bray GA, Clark JM, et al. Cardiovascular effects of intensive lifestyle intervention in type 2 diabetes. N Engl J Med. 2013;369:145–54.

2. CDC. Adult BMI Categories [Internet]. BMI. 2024 [cited 2024 Aug 25]. Available from: https://www.cdc.gov/bmi/adult-calculator/bmi-categories.html

3. American Diabetes Association Professional Practice Committee. 6. Glycemic targets: *standards of Medical Care in diabetes—2022*. Diabetes Care. 2022;45:S83–96.

4. CDC. Testing for Diabetes and Prediabetes: A1C [Internet]. Diabetes. 2024 [cited 2024 Sep 25]. Available from: https://www.cdc.gov/diabetes/diabetes-testing/prediabetes-a1c-test.html

5. Sherwani SI, Khan HA, Ekhzaimy A, Masood A, Sakharkar MK. Significance of HbA1c test in diagnosis and prognosis of diabetic patients. Biomark Insights. 2016;11:95–104.

6. Birtcher KK, Ballantyne CM. Measurement of cholesterol: A patient perspective. Circulation [Internet]. 2004 [cited 2024 Aug 25];110. Available from: https://www.ahajournals.org/doi/10.1161/01.CIR.0000141564.89465.4E

7. Urine albumin-creatinine ratio (uACR) [Internet]. National Kidney Foundation. [cited 2024 Aug 25]. Available from: https://www.kidney.org/kidney-topics/urine-albumin-creatinine-ratio-uacr

8. High Blood Pressure [Internet]. www.heart.org. [cited 2024 Aug 25]. Available from: https://www.heart.org/en/health-topics/high-blood-pressure

9. Cooper CC. Fundamentals of hand therapy: Clinical reasoning and treatment guidelines for common diagnoses of the upper extremity. St. Louis, MO: Mosby; 2006.

10. Lins L, Carvalho FM. SF-36 total score as a single measure of health-related quality of life: Scoping review. SAGE Open Med. 2016;4:2050312116671725.

11. Wirtz MA, Schulz A, Brähler E. Confirmatory and bi-factor analysis of the Short Form Health Survey 8 (SF-8) scale structure in a German general population sample. Health Qual Life Outcomes. 2021;19:73.

12. Lins-Kusterer L, Valdelamar J, Aguiar CVN, Menezes MS, Netto EM, Brites C. Validity and reliability of the 36-Item Short Form Health Survey questionnaire version 2 among people living with HIV in Brazil. Braz J Infect Dis. 2019;23:313–21.

13. Edelstein BA, Drozdick LW, Ciliberti CM. Assessment of depression and bereavement in older adults. Handbook of Assessment in Clinical Gerontology. Elsevier; 2010. p. 3–43.

14. Krantsevich N, He J, Richard Hahn P. Stochastic Tree Ensembles for Estimating Heterogeneous Effects [Internet]. arXiv [stat.ML]. 2022. Available from: http://arxiv.org/abs/2209.06998

15. Chipman HA, George EI, McCulloch RE. BART: Bayesian additive regression trees. aoas. 2010;4:266–98.

16. Hill J, Linero A, Murray J. Bayesian Additive Regression Trees: A Review and Look Forward. Annu Rev Stat Appl. 2020;7:251–78.

17. Wendling T, Jung K, Callahan A, Schuler A, Shah NH, Gallego B. Comparing methods for estimation of heterogeneous treatment effects using observational data from health care databases. Stat Med. 2018;37:3309–24.

18. He J, Hahn PR. Stochastic Tree Ensembles for Regularized Nonlinear Regression. J Am Stat Assoc. 2023;118:551–70.

19. Causal Inference using Bayesian Causal Forests [R package bcf version 2.0.2]. 2024 [cited 2024 Aug 31]; Available from: https://CRAN.R-project.org/package=bcf

Supplementary Table 1. Baseline Characteristics of Analytic Samples.

Characteristics, n (%)	Intervention (Intensive Lifestyle Intervention)	Control (Diabetes Support and Education)
Individuals, No	N = 2,309	N = 2,288
Age	58.73 (6.71)	59.06 (6.78)
Gender		
Male	952 (41%)	945 (41%)
Female	1,357 (59%)	1,343 (59%)
Race/Ethnicity		
White	1,539 (67%)	1,534 (67%)
African American / Black (not Hispanic)	383 (17%)	377 (16%)
Hispanic	300 (13%)	301 (13%)
Other/Mixed	87 (3.8%)	76 (3.3%)
Income		
<$10,000	78 (3.8%)	89 (4.3%)
$10,000-$19,999	147 (7.1%)	133 (6.5%)
$20,000-$29,999	215 (10%)	212 (10%)
$30,000- $39,999	218 (11%)	218 (11%)
$40,000-$49,999	218 (11%)	230 (11%)
$50,000-$59,999	210 (10%)	192 (9.3%)
$60,000- $69,999	182 (8.8%)	164 (8.0%)
$70,000-$79,999	174 (8.4%)	173 (8.4%)
>$80,000	630 (30%)	649 (32%)
Missing	237	228
Full Time Employment	1,155 (50%)	1,106 (49%)
Missing	7	13
Unemployed	476 (21%)	479 (21%)
Missing	6	9
Looking for Work	190 (8.2%)	164 (7.2%)
Missing	4	8
House Keeping/Rasing Children Full Time	724 (31%)	688 (30%)
Missing	5	11
Full/Part-Time Student	49 (2.1%)	72 (3.2%)
Missing	3	9
Family History of Diabetes	862 (37%)	773 (34%)
Number of Metabolic Syndrome Criteria Met	3.91 (0.95)	3.92 (0.91)
BMI	35.91 (6.00)	35.98 (5.77)
Smoking Status		
Never	1,126 (49%)	1,158 (51%)
Past	1,075 (47%)	1,034 (45%)
Present	104 (4.5%)	90 (3.9%)
Missing	4	6

Alcohol Consumption (oz/week)	8.90 (28.43)	8.70 (21.34)
History of CVD	338 (15%)	310 (14%)
History of Myocardial Infarction	142 (6.2%)	139 (6.1%)
Missing	5	1
History of Stroke	62 (2.7%)	51 (2.2%)
Missing	5	2
History of Coronary Artery Bypass Graft	108 (4.7%)	104 (4.5%)
Missing	4	2
History of Percutaneous Transluminal Coronary Angioplasty	145 (6.3%)	131 (5.7%)
Missing	4	3
History of Kidney Disease	161 (7.0%)	180 (7.9%)
Missing	4	1
History of Sleep Apnea	289 (13%)	270 (12%)
Missing	5	4
Hurt When Bed Covers Touch Skin	65 (2.8%)	63 (2.8%)
Missing	6	2
Muscle Cramps in Feet/Legs	803 (35%)	768 (34%)
Missing	5	2
History of Arthritis	954 (41%)	968 (42%)
Missing	5	4
History of Open Foot Sore	240 (10%)	231 (10%)
Missing	5	1
Fasting Glucose (mg/dl)	152.52 (44.86)	153.37 (45.97)
Missing	160	172
Hemoglobin A1c %	7.23 (1.12)	7.28 (1.18)
Missing	157	174
HDL Cholesterol (mg/dl)	43.39 (11.84)	43.39 (11.54)
Missing	160	172
LDL Cholesterol (mg/dl)	112.28 (32.01)	112.28 (32.16)
Missing	161	172
Triglycerides (mg/dl)	183.10 (113.86)	180.40 (114.71)
Missing	160	172
Serum Creatinine (mg/dL)	0.82 (0.20)	0.82 (0.20)
Missing	160	172
Urine Albumin (mg/dl)	4.79 (18.86)	5.19 (28.54)
Missing	163	175
Urine Creatinine (mg/dl)	120.89 (67.30)	119.14 (66.52)
Missing	213	225
Urine Albumin Creatinine Ratio	43.04 (187.57)	46.86 (230.99)
Missing	165	180
Systolic Blood Pressure	257.02 (34.52)	259.94 (34.62)
Missing	193	185
SF-36 General Health	47.12 (9.08)	47.31 (8.69)
Missing	6	9
SF-36 Transition	2.87 (0.81)	2.84 (0.79)

Missing	8	6
SF-36 Mental Component Summary	53.97 (8.03)	54.49 (7.72)
Missing	10	12
SF-36 Mental Health	53.57 (7.99)	53.96 (7.68)
Missing	6	7
SF-36 Bodily Pain	50.62 (8.69)	50.92 (8.61)
Missing	9	10
SF-36 Physical Component Summary	47.96 (7.91)	48.00 (7.94)
Missing	10	12
SF-36 Physical Functioning	48.61 (7.62)	48.54 (7.83)
Missing	6	6
SF-36 Role-Emotional	51.63 (7.72)	52.00 (7.12)
Missing	9	10
SF-36 Role-Physical	50.32 (8.10)	50.49 (7.78)
Missing	10	10
SF-36 Social Functioning	52.18 (7.67)	52.65 (7.06)
Missing	6	7
SF-36 Vitality	52.87 (9.20)	53.28 (8.76)
Missing	6	7
Beck Score of Depression	5.62 (5.14)	5.46 (4.56)
Missing	8	11

[a]Data are reported n (%) for categorical variables, mean (SD) for continuous variables.

Abbreviations: BMI, body mass index (kg/m²); CVD, cardiovascular disease; HDL, high density lipoprotein (mg/dL), LDL, low density lipoprotein (mg/dL); and SF-36, 36 Item Short Form.

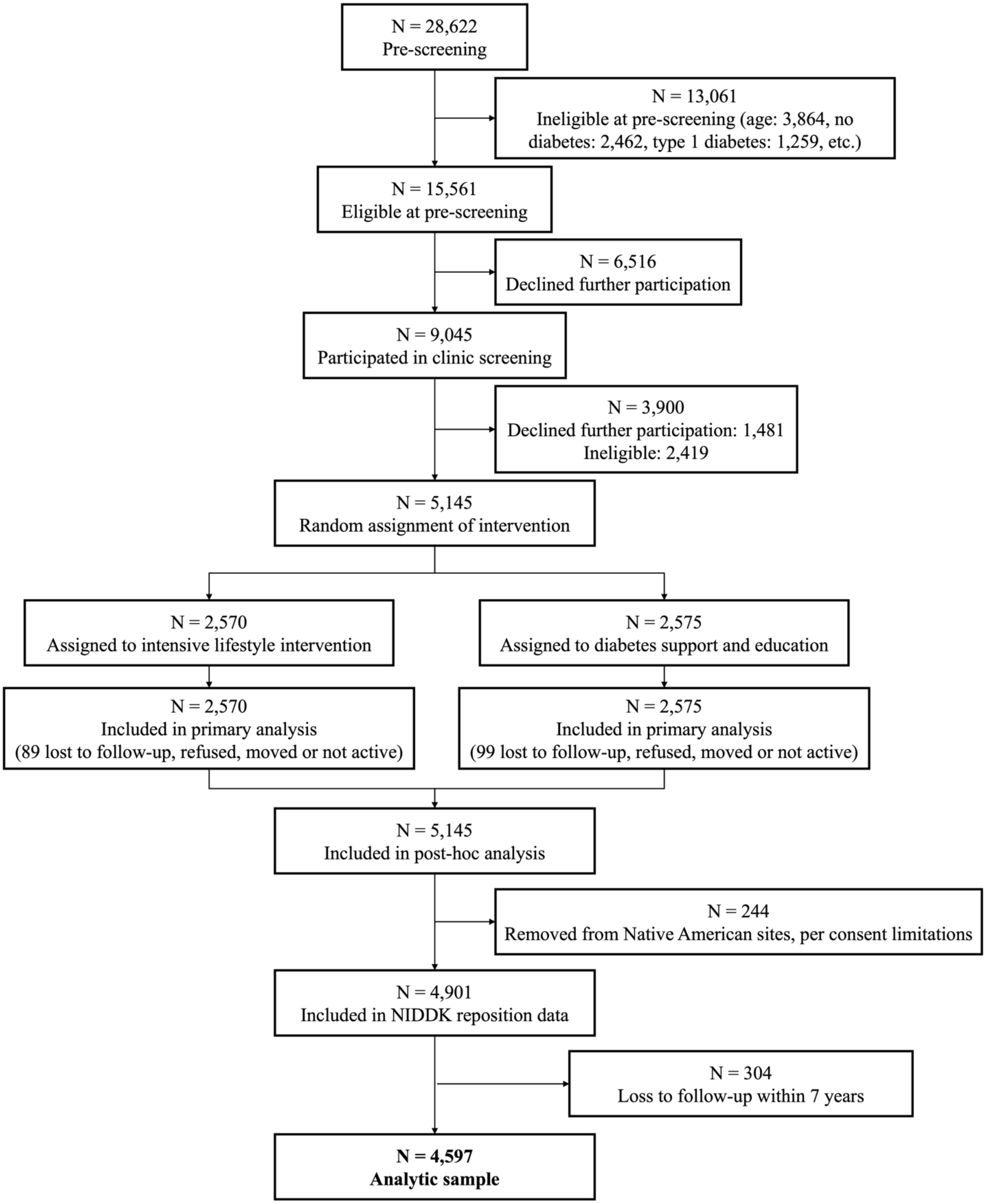

Supplementary Figure 1. Sample Flow Chart.
Abbreviations: NIDDK, National Institute of Diabetes and Digestive and Kidney Diseases.

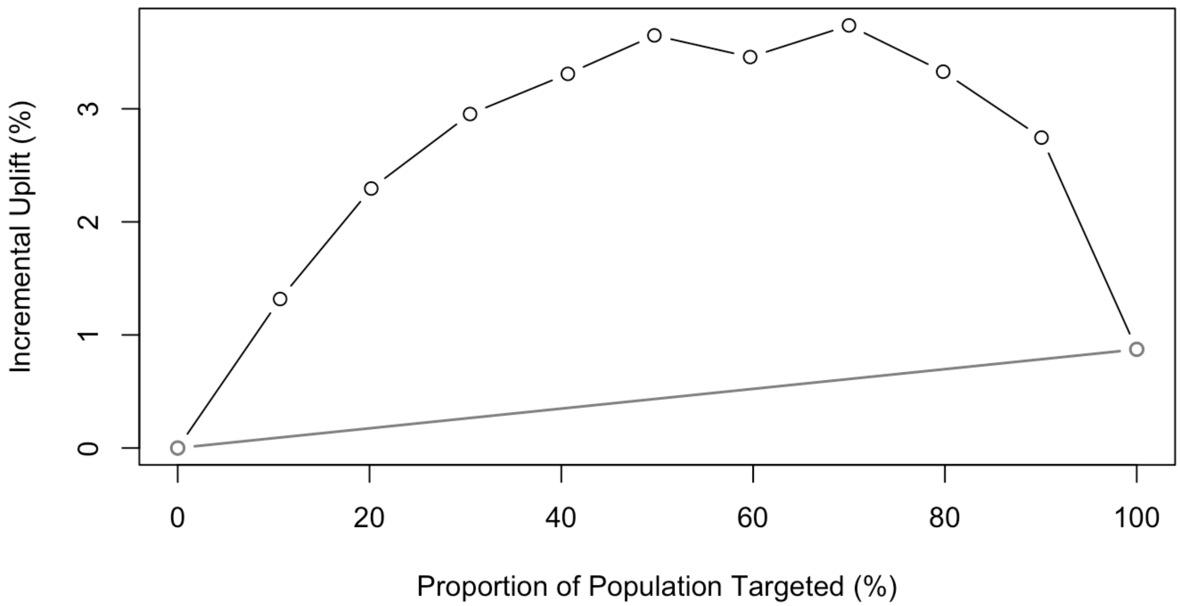

Supplementary Figure 2. The Qini curve.[a]

[a] The dotted line indicates the cumulative proportion of samples benefited from the intensive lifestyle intervention on the cardiovascular events after ordering samples from the lowest to highest conditional average treatment effects estimated via Bayesian causal forest. The solid line represents the cumulative proportion in a scenario where the ordering was conducted at random.

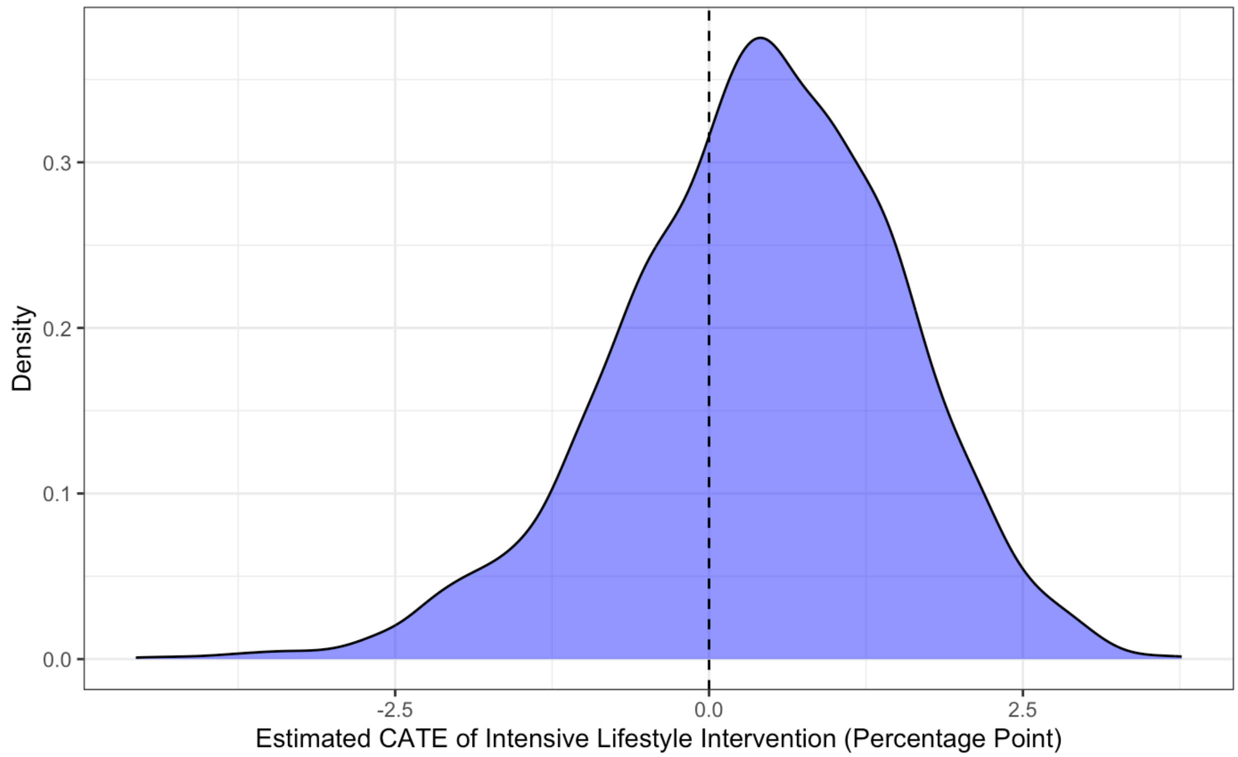

Supplementary Figure 3. Distribution of Conditional Average Treatment Effect of Intensive Lifestyle Intervention on Cardiovascular Events. [a]

Abbreviations: CATE, conditional average treatment effect.

[a] Conditional average treatment effects were estimated by the Bayesian causal forest algorithm.

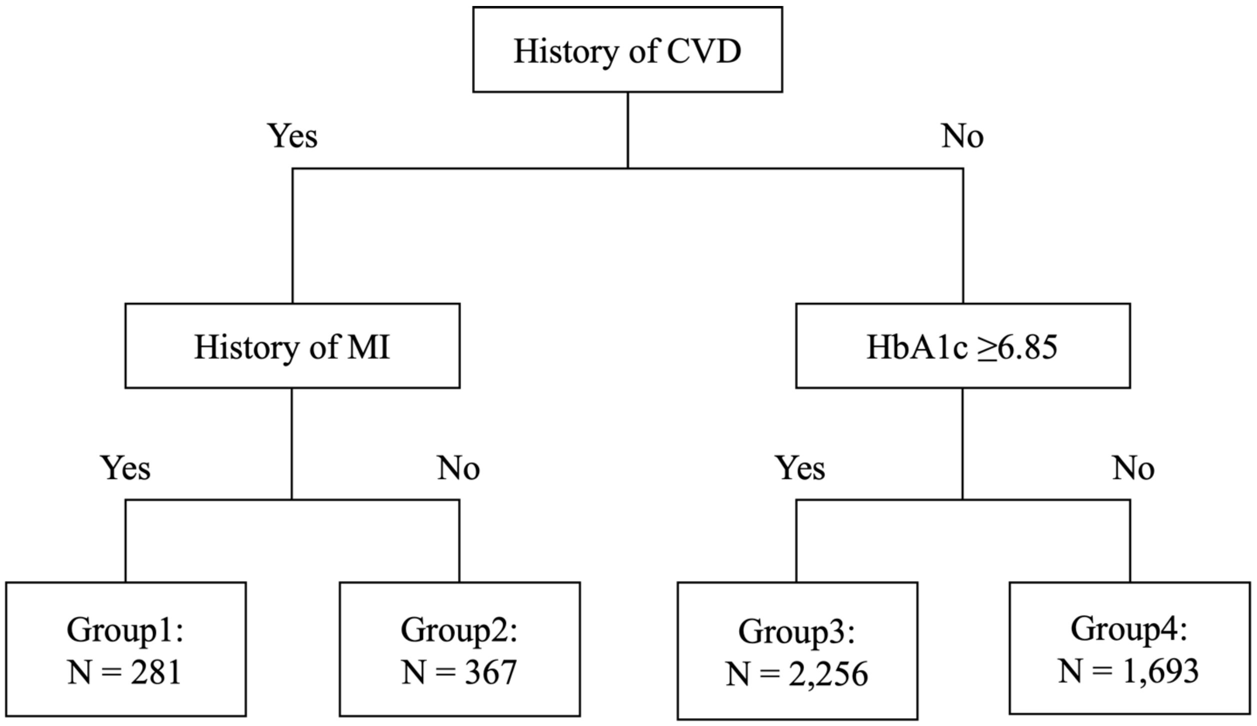

Supplementary Figure 4. Subgroup Discovery without Interpretable Covariates (All 47 Covariates).[a]

Abbreviations: CVD, cardiovascular disease; MI, myocardial infarction; and HbA1c, hemoglobin A1c (%).

[a] Classification and regression tree was created to predict the conditional average treatment effects using all 47 covariates. Conditional average treatment effects were estimated by the Bayesian causal forest algorithm.
